# Narrative pedagogy to promote health and wellbeing in school setting: an approach proposed by UNESCO chair on health education and sustainable development

**DOI:** 10.15171/hpp.2020.01

**Published:** 2020-01-28

**Authors:** Manuela Pulimeno, Prisco Piscitelli, Alessandro Miani, Salvatore Colazzo, Alfredo Mazza, Annamaria Colao

**Affiliations:** ^1^PhD Candidate in Human Relations Sciences, University of Bari “Aldo Moro”, Bari, Italy; ^2^UNESCO Chair on Health Education and Sustainable Development, University Federico II School of Medicine, Naples, Italy; ^3^Euro Mediterranean Scientific Biomedical Institute (ISBEM), Bruxelles, Belgium; ^4^Italian Society of Environmental Medicine (SIMA), Milan, Italy; ^5^Department of Environmental Science and Policy, University of Milan, Milan, Italy; ^6^Department of History, Society and Human Studies, University of Salento, Lecce, Italy

The scientific community has called for integrating the issue of young people’s health and wellbeing in all policies, starting from educational system.^1,2^ The United Nations sustainable development goals (SDGs) propose education as a crucial factor for improving individual quality of life, so that UNESCO is implementing a specific global strategy on “education for health and wellbeing”.^3^ Actually, school represents the ideal setting where unhealthy lifestyles and risk factors for chronic diseases can be addressed. Promoting students’ physical, emotional and social wellbeing has also an undoubtable effect on academic achievements. Thus, scholastic organizations, together with all the stakeholders involved, should systematically consider how educational approaches could increase students’ health awareness and critical thinking about unhealthy behaviours and their consequences. As UNESCO chair on Health Education and Sustainable Development, recently established at Federico II University of Naples, we recommend narrative-based interventions as highly-motivational educational strategy to promote wellbeing and healthy behaviours among students (paying special attention to pupils belonging to the most vulnerable social groups). Our minds spontaneously translate the experience into narrative terms as basic model of interpretation of reality (narrative thinking).^4^ Narration encourages students to think about thinking (metacognition), which allows young people to develop skills of judgment and interpretive thinking, resulting in applicable knowledge and responsible commitment to one’s own health (self-empowerment). Narrative pedagogy shifts from notions-centred approaches to meaningful learning, thus having the potential of enriching school curricula with transversal skills in the perspective of life-long learning. Teaching narratively consists in using stories (including digital storytelling) to provide positive models and deliver health contents^5^ in a joyful non-competitive environment, with techniques based on cooperative and experiential learning, role-playing and problem-solving.In our vision, narration represents the lever for spreading a culture of prevention and knowledge-based health promotion.

## Ethical approval

Not applicable.

## Competing interests

The authors declare that they have no competing interests.

## Authors’ contributions

MP, PP, AMi, SC, and AC conceived, wrote and revised this letter. AMa contributed to initially setting up the initiative.
